# Evaluating the parents taking action intervention among low-resourced Chinese immigrant families of children with autism: pre-, post-, and follow-up outcomes

**DOI:** 10.3389/fpsyt.2026.1774712

**Published:** 2026-08-07

**Authors:** Yue Xu, Weihai Zhan, Himani Garg, Hattie Gibson, Yuchen Chen

**Affiliations:** 1 Department of Health Sciences Education, University of Illinois Chicago College of Medicine, Chicago, IL, United States; 2 University of Illinois Chicago School of Public Health, Chicago, IL, United States; 3 Dell Medical School Department of Psychiatry & Behavioral Sciences, University of Pennsylvania, Philadelphia, PA, United States

**Keywords:** autism, culturally appropriate intervention, immigrant, parent-mediated intervention, self-efficacy

## Abstract

This study evaluates the changes over three different points of assessment on family and child outcomes of the Parents Taking Action (PTA) program among underserved Chinese immigrant families raising children with autism. Despite growing autism prevalence among Asian American children, Chinese immigrant families continue to face cultural, linguistic, and systemic barriers in accessing timely and effective services. The culturally-adapted Parents Taking Action-Chinese intervention was delivered virtually by trained community health workers (CHWs) with lived experiences. Twenty-eight parents were recruited from Chicago and New York City. Over a 10-week period, families participated in peer-led sessions covering evidence-based strategies (EBS), communication, play, advocacy, and coping. Family empowerment and caregivers’ self-efficacy in using EBS were maintained at the 2-week post-intervention. Notably, child outcomes—such as reductions in challenging behaviors, improvements in social communication, and increased access to clinical services—showed significant improvement at 3-month post-intervention, though they were not evident immediately post-intervention. These findings highlight the promise of scalable, community-based, and culturally responsive interventions to reduce disparities in autism care for Chinese immigrant families.

## Introduction

The population of Asian Americans and Pacific Islanders (AAPI) in the United States has more than doubled from 2000 to 2023, making them the fastest-growing ethnic and racial group (1). Within this population, the number of children diagnosed with autism has also drastically increased. Recent national surveillance data indicate a higher prevalence of autism among Asian children compared to non-Hispanic White children (2), and one large pediatric network study reported that Asian children had the highest rate of autism among all racial groups (3). Despite this growing prevalence, Asian American families remain markedly underrepresented in autism intervention research (4) and frequently experience inequitable access to medical, educational, and community-based supports and services (5, 6).

Importantly, AAPI families are not a monolithic group, and experiences of disability are shaped by immigration history, language access, socioeconomic status, and acculturation (7, 8). Chinese Americans—the largest AAPI subgroup and the focus of the present study (9)—often navigate autism care while simultaneously adapting to unfamiliar healthcare, educational, and social service infrastructures.

For many immigrant families, limited English proficiency, employment precarity, and constrained social networks further complicate the process of seeking timely diagnosis and evidence-based intervention for their children (10). As a result, disparities in autism care among Chinese families reflect not only cultural differences but also structural inequities embedded within service delivery systems.

Chinese immigrant families encounter a constellation of systemic, cultural, and societal barriers when seeking support for children with autism (11, 12). Systematic barriers include limited English proficiency, employment precarity, constrained social networks, and a shortage of culturally responsive and multilingual resources, as well as fragmented service systems and experiences of racial bias and discrimination (6, 10). For example, national studies have shown that Asian parents, compared to White parents, are less likely to report that healthcare providers offered culturally sensitive support, collaborative communication, or adequate information (6). These experiences can erode trust in service systems and contribute to delayed engagement with evidence-based interventions.

Cultural factors may further shape help-seeking and advocacy behaviors. Values such as deference to professional authority, stigma surrounding disability, and concerns about “saving face” -the cultural practice of maintaining social harmony, dignity, and family reputation by avoiding actions that could bring embarrassment or public scrutiny-may discourage families from openly discussing developmental concerns or challenging service providers (13). In addition, the pervasive “model minority” myth—which portrays Asian Americans as uniformly successful and self-sufficient—can obscure the very real struggles faced by low-resourced Chinese immigrant families raising children with developmental disabilities (4). Together, these intersectional obstacles have been associated with delayed diagnoses, reduced service utilization, disengagement from school systems, and heightened parental stress among Chinese families of children with autism (12, 14).

Despite these documented disparities, existing autism interventions rarely account for the cultural contexts of Chinese immigrant families. Many parent-mediated interventions are developed and tested primarily with English-speaking, middle-income families, and they presume familiarity with U.S. healthcare and educational systems, individualistic models of parent–provider interaction, and ready access to professional support (15). As a result, such interventions are often perceived by Chinese immigrant parents as overly clinical, linguistically inaccessible, and disconnected from their lived experiences (16). There remains a critical need for culturally responsive interventions that preserve core evidence-based components while explicitly addressing the realities of immigrant family life.

To date, relatively few parent psychoeducation interventions have been specifically developed for Chinese immigrant families of children with autism in the United States. One pilot study by Hsu-Min Chiang and colleagues implemented a parent education program with nine Chinese immigrant families in New York, reporting reductions in parental stress, increases in parental confidence, and improvements in select domains of parental quality of life, including physical health and environmental well-being (17). Notably, the intervention content in Chiang’s study was developed primarily on the basis of a parent interest survey rather than through a formalized cultural adaptation framework. While community-driven tailoring represents an important step toward culturally responsive programming, the use of structured frameworks such as the Ecological Validity Model (EVM) may provide additional methodological rigor by systematically addressing dimensions including language, context, metaphors, persons, and goals during adaptation (18).

To address disparities in autism service access among culturally and linguistically diverse families, the Parents Taking Action (PTA) program was originally developed as a culturally responsive parent-mediated intervention for Latinx families (19, 20). PTA integrates psychoeducation, naturalistic behavioral strategies, and facilitated peer discussion to enhance parental knowledge, self-efficacy, advocacy skills, and navigation of complex service systems. Delivered by trained community health workers with lived experience, the program emphasizes empowerment, social connection, and practical skill-building to support families facing structural barriers to care. Findings from the original PTA trial also suggest that improvements in parents’ outcomes may support the maintenance of child and family gains over time (19–21).

Building on this model, the present study evaluates the Parents Taking Action–Chinese program (PTA-Chinese), a culturally adapted version of PTA designed for Chinese immigrant families. PTA-Chinese was adapted using the Ecological Validity Model (18), incorporating linguistic, contextual, and procedural modifications based on feedback from Chinese immigrant parents (16). The adaptation process was community-informed and collaborative: Chinese parent caregivers participated in focus groups and were asked how the original PTA intervention should be modified to better fit the cultural values, language needs, family routines, stigma-related concerns, and service-system realities of Chinese immigrant families. Their feedback informed modifications to intervention language, delivery format, cultural examples, discussion topics, and procedures. The program is delivered by trained community health workers who are themselves Chinese immigrant mothers raising children on the autism spectrum. The intervention also included peer discussion and parent-guided goal setting, allowing parents to identify flexible goals based on their child’s needs and family priorities while receiving support from community health workers and the study team as needed.

## Conceptual framework and expected mechanisms of change

The present study is guided by a transactional model of child development, self-efficacy theory, and parent-mediated autism intervention frameworks. Transactional models conceptualize child development as emerging through repeated, bidirectional exchanges between the child and caregiving environment, rather than through child characteristics alone (22). In parent-mediated interventions, parents are not simply recipients of psychoeducation; they serve as the primary agents who embed intervention strategies within naturally occurring routines, including play, caregiving, communication, and community activities. From a self-efficacy perspective, parents’ confidence in their ability to support their child is expected to influence whether they initiate, persist in, and adapt intervention strategies when implementation is challenging (23). The original PTA is also guided by Bandura’s self-efficacy theory (19). This mechanism is especially relevant in autism interventions, where parental self-efficacy is linked to parents’ engagement in intervention, perceived competence, and the parent–child relationship (24). Empirical studies and reviews of parent-implemented interventions suggest that when parents learn to use responsive, naturalistic, and evidence-based strategies with greater confidence and fidelity, these strategies can improve the quality and frequency of parent–child interactions and create repeated opportunities for child communication, social engagement, and behavior regulation (25–28). Similarly, naturalistic developmental behavioral intervention models emphasize embedding developmentally appropriate strategies within naturally occurring interactions, following the child’s lead, using natural contingencies, and creating repeated opportunities for communication, play, and social engagement (29).

## Study aims and research questions

Prior studies of PTA-Chinese have demonstrated promising post-intervention gains in family empowerment, child access to services, as well as increased frequency of parents’ use of evidence-based strategies (16, 30). However, most intervention studies with immigrant and minoritized families rely on immediate post-intervention assessments, which may not capture the full trajectory of change. Repeated outcome assessments are especially important for low-resourced, minoritized immigrant families, who may require additional time to internalize new knowledge, practice skills, and overcome systemic barriers to implementation.

Accordingly, the current study extends prior work by examining family and child outcomes of PTA-Chinese across three time points: program baseline, post-intervention within two weeks of program completion (hereafter referred to as “post-intervention”), and 3-month post-intervention follow-up. The short-term goal of this study was to assess whether parent, family, and child outcomes showed improvement following participation in the intervention. The longer-term goal was to explore whether any observed changes were evident across a short follow-up period and whether patterns of change differed between parent/family outcomes and child outcomes. Findings from this work may help inform the design, evaluation, and dissemination of culturally responsive parent-mediated interventions aimed at reducing disparities in autism care. Specifically, the study addressed the following research questions:

1. Do family and parent outcomes (including parental self-efficacy, frequency of evidence-based strategy use, family empowerment, and parental stress) show significant change from baseline to post-intervention and 3-month follow-up?
2. Do child outcomes (including social communication, challenging behavior, adaptive functioning, and service utilization) show significant change over the same period?
3. Do temporal patterns of change differ over time, such that parent and family outcomes show earlier improvement than child outcomes?

## Methods

### Study design

The current study employed a single-arm, quasi-experimental design, with assessments collected at three time points: pre-intervention baseline, within 2 weeks post-intervention, three months post-intervention.

### Participants

The study was approved by the University of Illinois Chicago’s Institutional Review Board (Protocol #2019-0774) in September 2020. Participants were recruited from community organizations, online groups, and provider referrals in Chicago and New York City. These organizations served as dissemination channels only and were not research partners. They did not recruit or enroll participants directly; their role was limited to electronically sharing the study flyer. Inclusion criteria included (1) being born in China or another Chinese-speaking country/region, (2) fluency in Mandarin or Cantonese (reading, understanding, and speaking), and (3) serving as the parent and primary caregiver of an autistic child under 10 years old. Participants in this study were ethnically Chinese families living in the United States; all of them were immigrants, and some became naturalized US citizens. Because the intervention focuses on migration-related barriers such as language access and system navigation, we primarily use the term Chinese immigrant families when describing the study population. In contrast to the original PTA study, which recruited parents of children under age eight due to delayed diagnosis among children of color (19), the current study expanded the age criterion to under 10 years. This adjustment was made to address potential sampling challenges and to account for delayed diagnosis and barriers that Chinese immigrant parents may face in accessing autism-related knowledge and services in a timely manner.

Among the twenty-eight parents (mean age = 36.0 years, SD = 8.1) who participated in the study, 27 were mothers of children with autism, and one was a grandmother. Most of the participants were married or cohabiting (89%), and 57% were employed. The majority (71%) received their education outside the U.S., and 68% reported speaking limited or no English. Only 43% of the participants had completed education beyond the high school level, with only 29% reporting annual household income above $35,000. The average time lived in the U.S. was 10.8 years (SD = 5.7). See more details on participants demographic characteristics in **Table 1**.

**Table 1:** Participants demographics.

Characteristics	N (or n/N)	% or Mean (SD)
Child characteristics		
Child age in years	28	5.4 (2.1)
Child gender (male)	21/28	75.0%
Age first notice in months	28	20.1 (7.0)
Age of formal diagnosis in months	23	33.5 (13.2)
Parent characteristics		
Parent age in years	28	36.0 (8.1)
High school education or less	16/28	57.1%
Received education only in the home country	20/28	71.4%
Speak English Well or Very Well	9/28	32.1%
Employed	16/28	57.1%
Married or living together	25/28	89.3%
Years in the US	28	10.8 (5.7)
Good or excellent health	10/28	35.7%
Family characteristics		
Two or more children	5/28	17.9%
Three or more adults at home	13/28	46.4%
Income ≥ $35,000	8/28	28.6%

Children (mean age = 5.4 years, SD = 2.1, n=28) were predominantly male (75%). The mean age of the children when parents first noticed developmental concerns was 20.1 months (SD = 7.0). Mean age of formal diagnosis was 33.5 months (SD = 13.2).

### Intervention overview and community involvement

The Parents Taking Action program adapted for Chinese parents (PTA-Chinese) was delivered virtually over 10 weeks, including pre-recorded lectures and peer-led group discussions. The discussion leaders were four trained community health workers (CHWs)–Chinese immigrant mothers of youth and young adults on the spectrum. Three out of the four CHWs were identified through a nonprofit organization in Chicago that serves Chinese immigrant families of children with autism and other developmental disabilities. The other CHW was identified through the first author’s network of providers in New York. All CHWs received financial compensation for their time.

Major topics covered included understanding autism, communication, play, behavior strategies, and advocacy, with additional modules tailored to the cultural context (e.g., bilingualism myths, stigma, etc.). Each online group meeting lasted 60 minutes on average.

The intervention format was also adapted from one-on-one home visits to virtual group meetings to minimize participant burden and allow for easier participation (16). Families received bilingual materials (e.g., picture schedules, token boards) via mail and private access to session videos. They were asked to review pre-recorded lectures on YouTube prior to each week’s meeting.

Four CHWs (three from Chicago, one from New York) completed a structured 15-hour training curriculum and attended weekly supervision. Each CHW led a group of five to nine parents. Each session began with a brief check-in, mindfulness activity, and review of key concepts. Participants received a weekly assignment at the end of each session, which was to be completed and discussed in the following week.

The intervention also incorporated parent-guided individualized goal setting. During Week 3 of the 10-week program, parents were asked to identify individualized goals based on their child’s developmental needs, family priorities, and daily routines. These goals were intentionally flexible rather than prescriptive. Community health workers and the study support team were available to help parents refine goals, answer questions, and discuss how intervention strategies could be adapted to the family’s context. Some parents actively raised questions about how to apply strategies to their child’s needs, whereas others used the goals more independently. This process provided an opportunity for parents to move beyond the structured intervention content and to consider how PTA-Chinese strategies could be applied to their own child and family routines.

Detailed descriptions of the adaptation process and intervention procedures are available in previously published works (16, 30). Implementation findings from the same PTA-Chinese pilot, including parent engagement, attendance and retention, social validity, perceived cultural relevance, participant acceptability, focus group feedback, and community health worker delivery fidelity, have also been reported elsewhere (30).

### Measures

Family and child outcome measures were collected at three time points: baseline, post-intervention, and follow-up (3-month post-intervention). These outcomes were organized conceptually into three domains: intervention outcomes, implementation outcomes, and potential mediators/mechanism of change. Furthermore, because PTA-Chinese is a parent-mediated psychoeducational intervention, the primary intervention targets were parent- and family-level outcomes, including parental self-efficacy, use of evidence-based strategies, family empowerment, and parental stress. Child outcomes were included as exploratory and more distal indicators of change. This approach is consistent with prior PTA studies, in which the intervention was designed primarily to strengthen parents’ empowerment, confidence, and use of evidence-based practices, while child outcomes were examined as secondary outcomes (19, 20).

#### Intervention outcomes

Intervention outcomes included family-level and child-level outcomes. As mentioned above, PTA primarily supports parents rather than directly intervening with children, family-level outcomes were conceptualized as more proximal intervention outcomes, whereas child-level outcomes were conceptualized as distal intervention outcomes. Family- level intervention outcomes involve family empowerment and parental stress. Family empowerment was measured using the Family Outcome Survey-Revised (FOS; 31), while parental stress was measured using the Parenting Stress Index-Short Form (PSI-SF, 32). The FOS was translated and validated in Chinese by Poon et al. (33). In the current sample, Cronbach’s alpha was 0.89. Similarly, the PSI-SF was translated and validated with the Chinese population (34) and Cronbach’s alpha for the current sample was 0.86.

These family-level outcome measures were selected as parent outcomes because these constructs have been widely examined among Chinese immigrant families raising children with developmental disorders. Prior research indicates that parental self-efficacy is a culturally relevant mediator of parenting practices among immigrant families, with higher self-efficacy associated with strong adaptation to mainstream service systems and more supportive parenting behaviors (35). Similarly, Parental stress is another commonly studied construct among Chinese families of children with autism. (36) Additionally, family empowerment aligns with collectivist cultural values emphasizing relational well-being and family functioning. Yip (37) notes that culturally responsive empowerment approaches within Chinese communities may emphasize gradual, harmonious processes that strengthen both individuals and their family networks.

Child outcome measures included the Scales of Independent Behavior-Revised (SIB-R, 38) and the Social Communication Questionnaire (SCQ, 39). The SIB-R was translated and was previously tested in the Chinese population by Lin et al. (40). Cronbach’s alpha of the current sample was 0.83. Similarly, Gau and colleagues (41) translated and validated the Chinese version of the SCQ. The current study’s Cronbach’s alpha was 0.9.

#### Implementation outcomes

Implementation outcomes included parents’ uptake and use of intervention strategies. This domain was measured using parent-reported frequency of using evidence-based strategies in naturalistic routines. This was assessed using a 14-item measure developed by the original Parents Taking Action research team to evaluate how often parents used the intervention strategies taught in the curriculum (19). The first author, who is bilingual and bicultural, translated and back translated the items. Response options ranged from 1 = “never” to 4 = “always.” The measure demonstrated good internal consistency in the current sample, with a Cronbach’s alpha of 0.89.

#### Potential mechanisms of change

Parental self-efficacy and children’s access to evidence-based therapy/services were conceptualized as potential mechanisms of change. Parental self-efficacy was measured using a 10-item questionnaire created by the original PTA team (19). A four-point Lickert scale was used with 1= strongly disagree, 4= strongly agree. We summed parents’ responses. A higher score (up to 40) indicates higher levels of self-efficacy in using intervention strategies. For the current sample, Cronbach’s alpha was 0.84.

Another potential mechanism of change is children’s access to therapy/services. We tallied the total number of clinical services (occupational therapy, speech therapy, physical therapy, counseling, augmentative and alternative communication, applied behavioral analysis, and parent training) as well as social support services (parent support groups, social media groups, support from relatives, and special education advocates).

Regarding translation, all standardized measures (FOS, PSI-SF, SIB-R, SCQ) had been previously tested among Chinese families, and validated Chinese translations were available through purchase or open access. Measures assessing the number of clinical and social support services, parental self-efficacy, and the frequency of using evidence-based strategies were translated from English into Simplified Chinese and back-translated by the first author, who is bilingual and bicultural.

### Statistical analysis

Means and standard deviations were used to summarize participant characteristics (continuous) and outcomes at baseline, post intervention, and 3-month post-intervention. Categorial variables were summarized using numbers and percentages. Linear mixed models (LMM) using the SAS MIXED procedure accounted for correlations among repeated measurements (42). Based on the method of restricted maximum likelihood, the covariance structure was selected among three candidates: the compound symmetry (CS), first-order autoregressive (AR(1)), and unstructured (UN). Two commonly used information criteria, including Akaike’s information criterion (AIC) and the Bayesian information criterion (BIC), were used to select the covariance structure that showed the best fit. The error covariance structure with the smallest values of AIC and BIC was selected. If the AIC and BIC yielded inconsistent results, the parsimonious structure was selected. Only significant covariates were included in the model for adjustment; interactions between time and covariates were examined. Included covariates can be find in **Supplementary Table 1**. Multiple outcome variables were examined to identify individual outcomes that may be responsive to the intervention. Because these analyses were exploratory and the sample size was modest, p-values were not adjusted for multiple comparisons. Findings were therefore interpreted with emphasis on magnitude, direction of estimated changes, and the consistency of observed patterns rather than on statistical significance alone. In addition, *a priori*, a ≥10% change was defined as a pragmatic benchmark for potentially meaningful improvement and was used for descriptive interpretation rather than as a formal statistical threshold. Statistical significance was defined as p <.05. All statistical analyses were conducted using SAS version 9.4 (SAS Institute Inc).

## Results

Mean values of the outcomes at baseline, post-intervention, and Follow-up (3-month post-intervention) are summarized in **Table 2**.

**Table 2:** Means and standard deviations of outcomes at baseline, post-intervention, and 3-month follow-up (N = 28).

Child outcomes	Baseline	Post-intervention			3-Month follow-up	
		Mean (SD)	Cohen’s d	Mean (SD)		Cohen’s d
Social Communication (SCQ)	21.70 (5.84)	21.48 (7.48)	-0.03	19.23 (7.25)		-0.38
Social Interaction	10.33 (3.78)	9.44 (4.03)	-0.23	8.73 (4.60)		-0.38
Communication	5.26 (2.14)	6.07 (2.84)	0.32	5.46 (2.10)		0.10
Repetitive Behavior	5.00 (1.78)	4.74 (1.43)	-0.16	4.15 (1.78)		-0.48
Challenging behavior (SIB-R)	-20.52 (11.63)	-21.37 (12.76)	-0.07	-14.7 (11.8)		0.50
Number of supports and services						
# of clinical supports	1.93 (0.94)	2.15 (0.82)	0.25	2.70 (0.95)		0.82
# of social supports	4.54 (1.43)	3.96 (1.79)	-0.36	5.26 (1.63)		0.47
Evidence-based strategies						
Self-Efficacy in EBS	29.32 (6.28)	35.67 (3.95)	1.20	36.30 (3.44)		1.37
Frequency of using EBS	34.46 (7.52)	38.11 (6.92)	0.50	39.26 (7.02)		0.66
Family Empowerment (FOS)	78.14 (15.19)	85.48 (14.18)	0.50	85.30 (14.85)		0.48
Know Child’s Needs, Strengths	14.54 (3.38)	14.89 (3.07)	0.11	14.85 (3.08)		0.10
Know Child’s Rights	14.18 (4.12)	16.30 (4.02)	0.52	16.26 (3.91)		0.52
Know Child’s Development	12.32 (3.19)	13.59 (3.39)	0.39	13.30 (3.04)		0.31
Support Network	15.50 (4.26)	17.81 (4.78)	0.51	17.96 (4.74)		0.55
Community Participation	21.61 (4.92)	22.89 (4.29)	0.28	22.93 (4.21)		0.29
Parent Stress (PSI-SF)	108.4(16.01)	107.70 (16.59)	-0.04	106.78 (21.48)		-0.09

The effect size of Cohen's d was calculated as the difference between baseline mean score and mean score at corresponding follow-ups divided by the pooled standard deviation for each outcome.

Several parent and family outcomes showed significant improvements (≥10%) from baseline to 3-month post-intervention. Detailed temporal trends across outcomes, including percentage changes from baseline at within two weeks post-intervention and three-month post-intervention, are summarized in **Supplementary Table 2**.

### Family and parent outcomes

Family and parent outcome measures that showed significant and sustained improvement include: parental self-efficacy in using evidence-based strategies (EBS), frequency of EBS use, and multiple domains of family empowerment (knowing child’s rights, development, and building support network).

Parental self-efficacy in using EBS showed a 24% sustained improvement from baseline to 3-month post-intervention. Mean at baseline was 29.3 (SD = 8.1), at 2-week post-intervention was 35.7 (SD = 7.9), and maintained at 3-month post-intervention at 36.3 (SD = 8.2). The frequency of EBS use showed a 14% increase, which was maintained at the 3-month post-intervention.

### Exploratory parent-reported child outcomes

The study period saw a significant improvement in children’s social communication (social interaction and repetitive behavior subdomains) and a reduction in challenging behavior. Notably, the positive outcomes in both domains showed a delayed effect, with statistically significant improvement only observed at the 3-month post-intervention (not immediately post-intervention) after adjusting for relevant covariates (see **Table 3**). Additionally, the number of clinical supports increased from baseline (M = 1.93, SD = 1.2) to 2-week post intervention (M = 2.15, SD = 1.3) and again at 3-month post-intervention (M = 2.70, SD = 1.4), representing a 40% increase over the study period.

**Table 3:** Linear mixed models for outcomes (N = 28).

Outcomes	p-value of overall time	Post-intervention vs. baseline	3-Month follow-up vs. baseline
		Unadjusted model	
Social Communication (SCQ)	0.01	0.01 (0.89), p = 0.99	-2.35 (0.89), p = 0.01
Social Interaction	0.02	-0.70 (0.51), p = 0.17	-1.49 (0.51), p = 0.01
Communication	0.11	0.86 (0.42), p = 0.05	0.20 (0.42), p = 0.64
Repetitive Behavior	0.09	-0.26 (0.32), p = 0.41	-0.85 (0.39), p = 0.04
Challenging behavior (SIB-R)	0.04	-0.85 (1.95), p = 0.67	5.81 (2.21), p = 0.01
Number of supports and services			
# of clinical supports	<.0001	0.22 (0.20), p = 0.28	0.78 (0.16), p <.0001
# of social supports	<.0001	-0.52 (0.22), p = 0.03	0.78 (0.15), p <.0001
Evidence-based strategies			
Self-Efficacy in EBS	<.0001	6.28 (0.95), p <.0001	6.93 (0.99), p <.0001
Frequency of using EBS	<.0001	3.60 (0.84), p <.0001	4.76 (1.13), p <.001
Family Empowerment (FOS)	<.001	6.94 (1.68), p <.001	6.75 (1.68), p <.001
Know Child’s Needs, Strengths	0.87	0.28 (0.57), p = 0.63	0.24 (0.57), p = 0.67
Know Child’s Rights	<.0001	2.05 (0.43), p <.0001	2.01 (0.43), p <.0001
Know Child’s Development	0.02	1.21 (0.42), p <.01	0.92 (0.55), p = 0.10
Support Network	<.001	2.15 (0.58), p <.001	2.30 (0.58), p <.001
Community Participation	0.11	1.29 (0.71), p = 0.08	1.33 (0.71), p = 0.07
Parent Stress (PSI-SF)	0.76	-0.90 (1.76), p = 0.61	-1.81 (2.41), p = 0.46
		Adjusted Model	
Social Communication (SCQ)	0.03	0.08 (0.89), p = 0.93	-2.08 (0.90), p = 0.02
Social Interaction	0.02	-0.70 (0.51), p = 0.17	-1.49 (0.51), p = 0.01
Communication	0.08	0.89 (0.38), p = 0.02	0.37 (0.39), p = 0.35
Repetitive Behavior	0.03	-0.32 (0.30), p = 0.29	-1.00 (0.38), p = 0.01
Challenging behavior (SIB-R)	0.04	-0.85 (1.95), p = 0.67	5.81 (2.21), p = 0.01
Children's support and services			
# of clinical supports	<.0001	0.22 (0.20), p = 0.28	0.78 (0.16), p <.0001
# of social supports	<.0001	-0.52 (0.22), p = 0.03	0.78 (0.15), p <.0001
Evidence-based strategies			
Self-Efficacy in EBS	<.0001	6.18 (0.94), p <.0001	6.82 (0.97), p <.0001
Frequency of using EBS	<.0001	3.58 (0.84), p <.0001	4.72 (1.12), p <.001
Family Empowerment (FOS)	<.001	6.91 (1.68), p <.001	6.72 (1.68), p <.001
Know Child’s Needs, Strengths	0.87	0.28 (0.57), p = 0.63	0.24 (0.57), p = 0.67
Know Child’s Rights	<.0001	2.05 (0.43), p <.0001	2.01 (0.43), p <.0001
Know Child’s Development	0.02	1.21 (0.42), p <.001	0.92 (0.55), p = 0.10
Support Network	<.001	2.15 (0.58), p <.001	2.30 (0.58), p <.001
Community Participation	0.13	1.26 (0.71), p = 0.08	1.30 (0.71), p = 0.07
Parent Stress (PSI-SF)	0.73	-0.96 (1.76), p = 0.59	-1.91 (2.39), p = 0.43

Unless specified, data presented were regression coefficients of the difference (standard error); in the adjusted model, only significant characteristics were included in the model for adjustment.

**Table 3** presents unadjusted and adjusted means, along with standard errors and linear mixed model statistics. Comparing adjusted means at time 2 and time 3, exploratory parent-reported child indicators showed stronger evidence of change at 3-month follow-up (11) than immediately post-intervention (7).

## Discussion

This study examined the repeated outcomes of PTA-Chinese, a culturally adapted version of Parents Taking Action, among low-resource Chinese immigrant families raising children with autism. Overall, findings indicate sustained and, in some domains, growing improvements across the six-month study period, highlighting the potential durability of culturally responsive, parent-mediated interventions.

### Improving parental skills and confidence

Parental self-efficacy, frequency of EBS use, and multiple domains of family improved by more than 10% at the 2-week post-intervention, with gains maintained or further strengthened at 3-month post-intervention. These patterns are consistent with self-efficacy theory and conceptual models of parent-mediated intervention that position parental capacity, confidence, and strategy use as proximal targets through which child outcomes may improve over time. From a self-efficacy perspective, parents who feel more confident in their ability to support their child may be more likely to initiate, persist in, and adapt evidence-based strategies during everyday routines, thereby creating repeated opportunities for child communication, social engagement, and behavior regulation. However, the current study was not designed to formally test mediation pathways, and therefore causal inferences regarding the relationship between parent and child outcomes cannot be made. For immigrant families navigating linguistic barriers, limited service availability, and unfamiliar service systems, strengthening parents’ confidence and practical skills may be particularly important.

Importantly, PTA-Chinese was delivered in families’ primary language and facilitated by trained Chinese community health workers, which may have enhanced trust, relevance, and sustained engagement. While causal inference cannot be drawn, the durability of parent-reported improvements suggests that culturally and linguistically aligned intervention delivery may support longer-term uptake of strategies beyond the active intervention period.

The outcome findings should be interpreted alongside the published feasibility/acceptability findings, which showed high engagement, favorable social validity/cultural relevance ratings, and high delivery fidelity (30).

### “Emerging patterns in exploratory parent-reported child outcomes”

In contrast to parent outcomes, behavior exploratory parent-reported indicators of child social communication, challenging behavior, and access to clinical supports showed modest change at the 2-week post-intervention and stronger evidence of change at 3-month post-intervention. This temporal pattern is consistent with the possibility that parent-mediated changes may take time to appear in child-related outcomes; however, this interpretation remains tentative. For example, Green et al. (43), in an RCT with infant siblings at elevated likelihood of autism, observed a non-significant delayed trend toward improvement in child developmental outcomes, including Mullen language and Vineland communication scores. Similarly, Hampton et al. (44), using a single-subject design with preschool children receiving the RUBI intervention, reported meaningful reductions in disruptive behavior for two of three participants primarily during the later phases of treatment. In addition, Magaña et al. (2022) found that children’s access to services continued to improve at follow-up compared to post-intervention, suggesting that parent-mediated changes may translate into downstream gains over time. Together, these findings suggest that child-related changes in parent-,mediated interventions may sometimes emerge over time, consistent with the trajectory observed in the current PTA Chinese trial (21, 43, 44). Such temporal sequencing is theoretically plausible, as parents may require time to internalize new strategies, adapt them to their child’s needs, and implement them consistently within daily routines before measurable changes in the child emerge. This interpretation is also consistent with parent-mediated and NDBI-related frameworks, in which repeated caregiver–child interactions in natural routines are expected to create cumulative opportunities for child learning (25, 29). However, the present study cannot determine whether changes in parental self-efficacy or strategy use statistically mediated changes in child outcomes.

For low-resource immigrant families, this process may take additional time due to competing demands, limited formal supports, and structural barriers to services. These findings underscore the importance of aligning evaluation timelines with expected mechanisms of change rather than relying solely on immediate post-intervention assessments.

### Implications

The observed patterns have several implications for the design and evaluation of parent-mediated interventions, particularly those targeting underserved populations. Although the follow-up period in the current study was relatively brief, the observed trends suggest that future evaluations of parent-mediated interventions may benefit from including longer follow-up periods alongside more proximal and change-sensitive child outcome measures. Incorporating longer follow-up periods may provide a more accurate understanding of change trajectories. Second, intervention models may benefit from explicitly building in opportunities for ongoing practice, reinforcement, or low-intensity follow-up supports to help families consolidate skills over time. Given that most analyses did not reach statistical significance, these implications should be interpreted as preliminary and hypothesis-generating rather than confirmatory.

More broadly, the findings contribute to a growing literature emphasizing the importance of cultural adaptation—not only in language translation, but also in delivery format, facilitator characteristics, and responsiveness to family context. While effect sizes were modest and not consistently statistically significant, PTA-Chinese provides an example of how community-based, culturally aligned parent training can be implemented in ways that are accessible to immigrant families who are traditionally underrepresented in intervention research.

### Limitations and future directions

Several limitations should be noted. First, the sample size was modest (N = 28), which may limit the statistical power and generalizability of the findings. Second, the follow-up period extended only to three months post-intervention; therefore, conclusions regarding longer-term maintenance of effects should be interpreted with caution. Additionally, the absence of a control group limits causal inferences, although the use of repeated measures and observed patterns of change over time strengthens the interpretation of intervention-associated changes. Self-reported measures, while appropriate for capturing parent perspectives, may be influenced by social desirability or recall bias. Furthermore, child outcomes were based on parent reports rather than direct observational assessments. Child outcome measures, SCQ and SIB-R may have limited sensitivity to detect short-term intervention-related change. Thus the observed pattern is best understood as preliminary and hypothesis-generating.

Additionally, while the sample was described as low-resourced based on indicators such as household income, educational attainment, English proficiency, and service access, the study was not powered to examine how these factors influenced intervention response. The modest sample size limited our ability to conduct subgroup or moderation analyses by socioeconomic status, language proficiency, or baseline service access. Therefore, low-resource status should be interpreted as an important contextual characteristic of the sample rather than as an analytically tested predictor of outcomes. Future studies with larger samples should examine whether socioeconomic status, language proficiency, immigration-related barriers, and baseline service access moderate intervention engagement, implementation, and family or child outcomes.

Furthermore, temporal patterns suggested that improvements in parent outcomes preceded some child-level changes; however, the study was not powered or designed to evaluate mediation effects. Future studies using larger samples, observational measures of parent-child interaction, and formal mediation analyses are needed to clarify the mechanisms through which culturally adapted parent-mediated interventions may influence child outcomes.

Finally, although PTA-Chinese included parent-guided individualized goal setting during Week 3 and opportunities for parents to seek support from community health workers and the study team, this process was flexible and not systematically measured as an implementation outcome. The study did not collect detailed data on the content of each family’s goals, the extent to which goals were revised over time, or variation in parents’ use of individualized support. Future studies may strengthen the family-centered approach by incorporating more structured documentation of parent-selected goals, shared decision-making processes, and individualized strategy adaptation. Future work may also explore moderators of response, such as baseline parental stress, child developmental profile, or service access, as well as implementation outcomes, including acceptability, feasibility, and scalability, across diverse community settings.

## Conclusion

Our findings provide preliminary evidence that participation in a culturally adapted, community-based intervention, such as PTA Chinese, is associated with sustained improvements in both family outcomes along with possible changes in exploratory parent-reported child indicators over time. While early changes were primarily observed in family/parent outcomes, exploratory parent-reported child outcomes showed possible changes over time. The findings highlight the importance of culturally responsive intervention approaches and extended follow-up in understanding how change unfolds over time. PTA-Chinese shows promise as a scalable model for supporting families of children with autism and addressing disparities in access to evidence-informed support.

## Data Availability

The original contributions presented in the study are included in the article/**Supplementary Material**. Further inquiries can be directed to the corresponding author.
