# Supplementary material for: Evaluating the parents taking action intervention among low-resourced Chinese immigrant families of children with autism: pre-, post-, and follow-up outcomes
Source: Front Psychiatry. 2026 Aug 7;17:1774712. doi: 10.3389/fpsyt.2026.1774712 (PMC13495679; doi:10.3389/fpsyt.2026.1774712)
Supplement: Supplementary file 1 [file Table1.docx]

| **Supplementary Table 1. Covariates Included in Adjusted Models (Table 3)** | |
| --- | --- |
| Outcomes | Covariates Included in Model |
| Social Communication (SCQ) | Total English score |
| Social Interaction | Total English score |
| Communication | Total English score |
| Repetitive Behavior | Parent health |
| Challenging behavior (SIB-R) | No significant covariates; model unadjusted. |
| Number of supports and services | |
| Number of clinical supports | No significant covariates; model unadjusted. |
| Number of social supports | No significant covariates; model unadjusted. |
| Evidence-based strategies |  |
| Self-Efficacy in EBS | Income |
| Frequency of using EBS | Total English score; Education |
| Family Empowerment (FOS) | Education |
| Know Child’s Needs, Strengths | Total English score |
| Know Child’s Rights | Total English score; Education; Child gender |
| Know Child’s Development | Total English score |
| Support Network | Parent accepted education only in home country; Child gender |
| Community Participation | Education |
| Parent Stress (PSI-SF) | Three or more adults at home; English fluency |

| **Supplementary Table 2. Changes from Baseline (%) in Outcomes** | | |
| --- | --- | --- |
| Outcomes | 3-Month | 6-Month |
| Social Communication (SCQ) | -1.0% | -11.4% |
| Social Interaction | -8.6% | -15.5% |
| Communication | 15.4% | 3.8% |
| Repetitive Behavior | -5.2% | -17.0% |
| Challenging behavior (SIB-R) | 4.1% | -28.4% |
| Number of clinical supports | 11.4% | 39.9% |
| Number of social supports | -12.8% | 15.9% |
| Evidence-based strategies |  |  |
| Self-Efficacy in EBS | 21.7% | 23.8% |
| Frequency of using EBS | 10.6% | 13.9% |
| Family Empowerment (FOS) | 9.4% | 9.2% |
| Know Child’s Needs, Strengths | 2.4% | 2.1% |
| Know Child’s Rights | 15.0% | 14.7% |
| Know Child’s Development | 10.3% | 8.0% |
| Support Network | 14.9% | 15.9% |
| Community Participation | 5.9% | 6.1% |
| Parent Stress (PSI-SF) | -0.7% | -1.5% |
